# Cleavage of the selective autophagy receptor SQSTM1/p62 by the SARS-CoV-2 main protease NSP5 prevents the autophagic degradation of viral membrane proteins

**DOI:** 10.1186/s43556-022-00083-2

**Published:** 2022-06-03

**Authors:** Yabin Zhang, Shiyan Liu, Qingjia Xu, Huihui Li, Kefeng Lu

**Affiliations:** Department of Neurosurgery, State Key Laboratory of Biotherapy, West China Hospital, Sichuan University, Chengdu, 610041 China

**Keywords:** Autophagy, SQSTM1/p62, SARS-CoV-2, NSP5, Autophagic degradation

## Abstract

Severe acute respiratory syndrome coronavirus 2 (SARS-CoV-2) has caused the coronavirus disease 2019 (COVID-19) global pandemic. Omicron, a new variant of SARS-CoV-2, has the characteristics of strong transmission and pathogenicity, short incubation period, and rapid onset progression, and has spread rapidly around the world. The high replication rate and intracellular accumulation of SARS-CoV-2 are remarkable, but the underlying molecular mechanisms remain unclear. Autophagy acts as a conservative cellular defence mechanism against invading pathogens. Here, we provide evidence that the main protease of SARS-CoV-2, NSP5, effectively cleaves the selective autophagy receptor p62. NSP5 targets p62 for cleavage at glutamic acid 354 and thus abolishes the capacity of p62 to mediate selective autophagy. It was further shown that p62 specifically interacted with ubiquitinated SARS-CoV-2 M, the viral membrane protein, to promote its autophagic degradation. In the presence of NSP5, p62-mediated autophagic degradation of the M protein was inhibited. The cleaved products of p62 also cannot facilitate the degradation of the M protein. Collectively, our findings reveal that p62 is a novel host target of SARS-CoV-2 NSP5 and suggest that selective autophagy targets viruses and potential strategies by which the virus evades autophagic clearance. Our results may provide new ideas for the development of anti-COVID-19 drugs based on autophagy and NSP5.

## Introduction

The ongoing coronavirus disease 2019 (COVID-19) by the severe acute respiratory syndrome coronavirus 2 (SARS-CoV-2) in December 2019, has exerted a profound impact on healthcare systems and national economies worldwide [1–3]. Recently, a new SARS-CoV-2 variant named Omicron, B.1.1.529, has been reported in many countries, including China, and has led to a rapid increase in COVID-19 cases [4]. The Omicron variant has evolved more mutations on multiple proteins than the Alpha and Delta variants of SARS-CoV-2, such as NSP3, NSP4, NSP5, NSP6, NSP12, NSP14, S protein, E protein, N protein and M protein and it may be more threatening than the Alpha and Delta variants [5, 6]. The Omicron variant contain 36 mutations in the spike protein, therefore Omicron variant may have evolved the ability to spread more easily in the human population and resist currently available treatment antibodies to COVID-19 [7]. SARS-CoV-2 is a single-stranded positive sense RNA virus that contains multiple open reading frames (ORFs) [8]. The SARS-CoV-2 encodes pp1a (~ 450 kDa) and pp1ab (~ 750 kDa) [9, 10]. Both pp1a and pp1ab are cleaved into 16 nonstructural proteins by papain-like protease (PL^pro^) and main protease (M^pro^) to assemble the viral replicase complex [9, 11]. M^pro^, which is also known as 3C protease-like protease (3CL^pro^) or NSP5, releases NSP4-NSP16 by cleaving 11 conserved cleavage sites in pp1a and pp1ab to facilitate the formation of the viral replicase complex [12, 13]. NSP5 is a cysteine protease that recognizes a sequence containing Leu and Gln at P2 and P1 positions [14]. A catalytic active site of NSP5 is formed by His41 and Cys145 [15, 16]. In addition to conserved proteolytic cleavage sites in the viral polyproteins, very few cleavage sites have been identified in host cell proteins [17, 18].

Autophagy is an intracellular catabolic process mediated by the formation of double-membraned autophagosomes that transport intracellular substances for degradation, which maintains cellular metabolic balance and homeostasis [19–21]. Autophagy has been considered as nonselective [22]. However, ample evidence indicates that certain forms of autophagy are highly selective [23, 24]. SQSTM1/p62 (hereafter referred to as p62) is a signalling hub of diverse cellular activities, such as amino acid sensing, the NF-κB pathway and the type I IFN production pathway [25–27]. p62 has also been proposed to be a cargo receptor that contributes to the selective autophagy [24, 28]. p62 interacts with ubiquitinated substrates via its UBA domain (ubiquitin-associated domain), multimerizes via its PB1 domain (NH2-terminal Phox and Bem1p domain), and recruits cargoes to autophagosomes by binding with microtubule-associated protein light chain 3 (LC3) via the LC3-interacting-region (LIR) motif [29, 30]. p62 directly binds to the Sindbis virus capsid protein, Chikungunya virus capsid protein and Seneca Valley virus capsid protein and then targets these viruses for autophagic degradation [31–33]. However, it is unknown whether or how selective autophagy and the receptor p62 act on SARS-CoV-2 and how they are affected by SARS-CoV-2-encoded proteins.

Here, p62 was identified as a host cleavage target of NSP5 at Gln354, which impaired p62 as a receptor of selective autophagy. p62 interacted with SARS-CoV-2 capsid membrane protein M and targeted it for autophagic degradation. These data uncovered that SARS-CoV-2 impairs selective autophagy in host cells, which can be exploited for the development of antiviral drugs against COVID-19.

## Results

### SARS-CoV-2 NSP5 does not affect the autophagy machinery

**Fig. 1:**
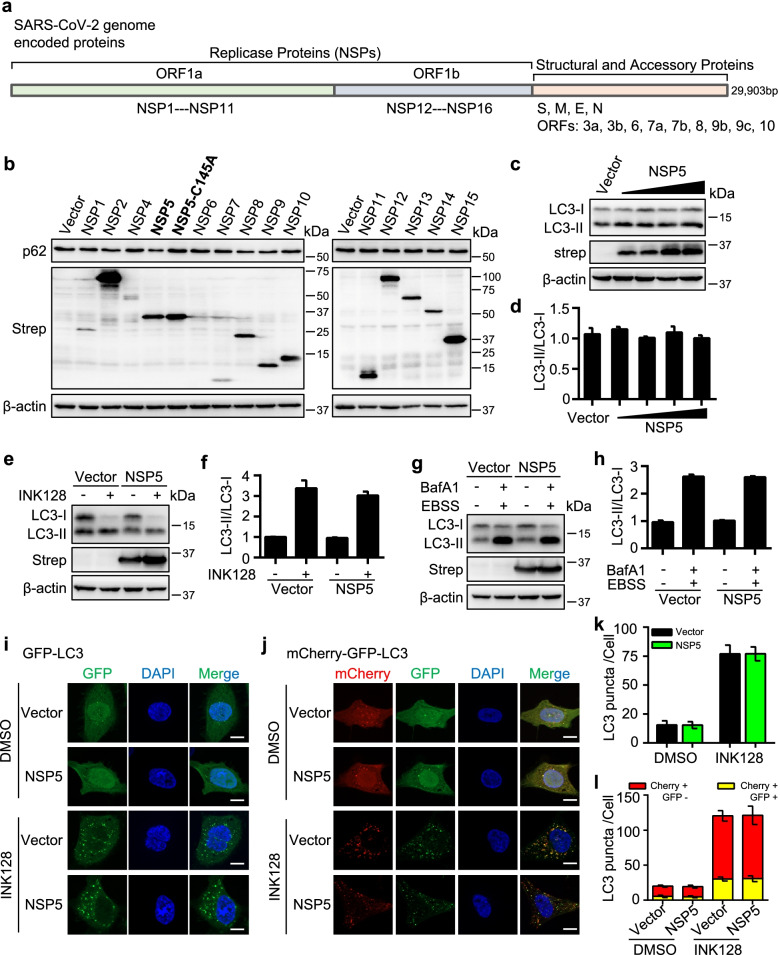
SARS-CoV-2 NSP5 cannot induce autophagy. **a** Scheme of SARS-CoV-2 genome and encoded proteins. **b** SARS-CoV-2-encoded non-structural proteins were screened for their effects on p62 in HEK293T cells. **c-d** SARS-CoV-2 NSP5 did not affect autophagy at basic conditions. The protein conversion from LC3-I to LC3-II in HEK293T cells with increased expression of NSP5 were analyzed (**c**) and quantified (**d**). **e–f** SARS-CoV-2 NSP5 did not affect autophagy. NSP5 was expressed in HEK293T cells with or without INK128 treatment and LC3-II/LC3-I ratios were observed (**e**) and quantified (**f**). **g-h** SARS-CoV-2 NSP5 cannot inhibit autophagy in autophagy-blocked cells. NSP5 was expressed in HEK293T cells followed by short-term treatment with EBSS starvation combined with Bafilomycin A1(BafA1) treatment and LC3-II/LC3-I ratios were observed (**g**) and quantified (**h**). **i, k** NSP5 did not affect autophagosome formation. Scale bars, 10 μm. **j, l** NSP5 did not affect autophagic flux. NSP5 was expressed in HeLa cells with stable expression of mCherry-GFP-LC3. Puncta of matured autolysosome formed after autophagosome-lysosome fusion (red puncta) and autophagosomes before fusion (yellow puncta) were observed (**j**) and counted (**l**). Scale bars, 10 μm

Our previous study showed that NSP5 significantly reduced the protein levels of p62 in HEK293T cells compared with the enzymatic mutant NSP5-C145A [34]. We hypothesized that NSP5 was involved in regulating the autophagic degradation of p62. To explore the effect of NSP5 and other nonstructural proteins encoded by SARS-CoV-2 on p62, we expressed these nonstructural proteins (except for NSP3 and NSP16) in HEK293T cells to determine their effects on p62 (Fig. 1a, b). We found that wild-type (WT) NSP5 significantly reduced p62 protein levels compared with the NSP5-C145A mutant (Fig. 1b). Since p62 is also degraded when it acts as an autophagy receptor, a reduction in p62 protein levels usually indicates autophagy activation. We hypothesized that NSP5 may be involved in regulating autophagy. We then expressed NSP5 and examined its effects on the autophagy marker LC3-II, which is converted from LC3-I upon autophagy induction (Fig. 1c). No significant alteration in the amount of LC3-II was detected in NSP5-overexpressing cells (Fig. 1c, d). We then determined the effect of NSP5 in response to autophagy stimulation (INK128 treatment; INK128, an effective autophagy activating chemical through inhibiting mTOR) or autophagy blockade (short-term EBSS starvation combined with BafA1 treatment), and the LC3-II levels showed that autophagy was not affected by NSP5 (Fig. 1e-h). GFP-LC3 puncta in cells treated with or without INK128 was similar to that in cells expressing NSP5 (Fig. 1i, k). We used mCherry-GFP-LC3 to examine autophagic flux-based differences between autophagosomes and autolysosomes. NSP5 expression did not affect the number of mature autolysosomes (red puncta) nor immature autophagosomes (yellow puncta) (Fig. 1j, l). Thus, the effect of NSP5 on p62 was thought to be independent of the autophagy machinery.

### SARS-CoV-2 NSP5 cleaves p62 through its protease activity

**Fig. 2:**
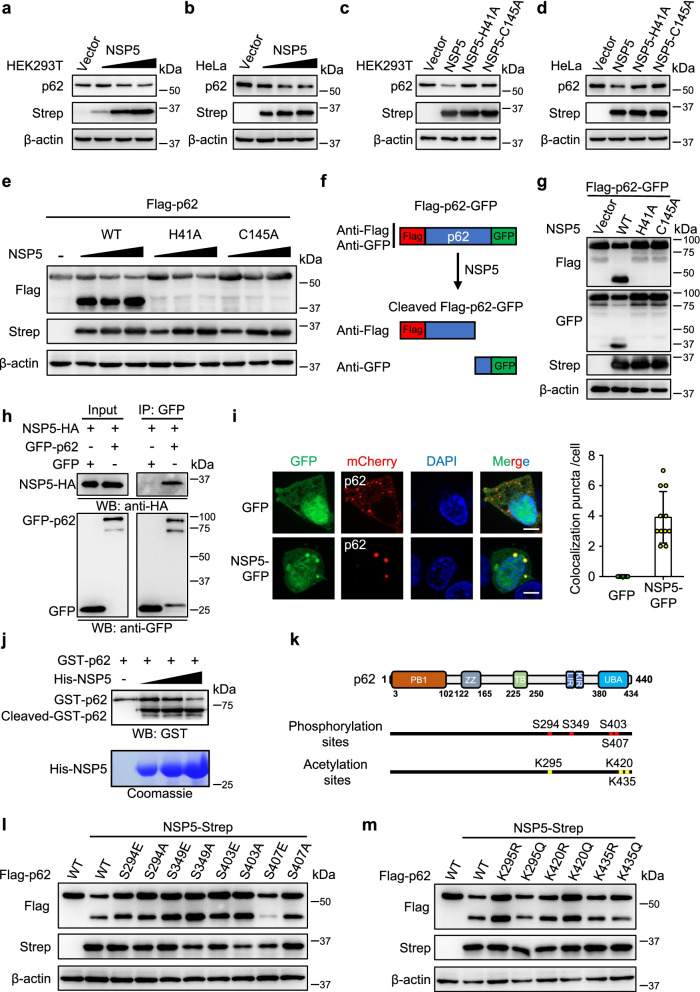
SARS-CoV-2 NSP5 targets p62 for cleavage through its protease activity. **a-b** NSP5 reduced the protein levels of p62. **c-d** HEK293T and HeLa cells were transfected with wild-type NSP5 or its enzymatic inactive mutants (H41A and C145A) for 48 h and then endogenous p62 was analyzed. **e** Flag-p62 were transfected with WT NSP5 or its enzymatic inactive mutants (H41A and C145A) into HEK293T cells for 48 h. **f** Schematic representation of Flag-p62-GFP cleaved by SARS-CoV-2 NSP5. **g** Immunoblotting analysis of ectopically expressed Flag-p62-GFP in HEK293T cells with the expression of WT NSP5 or its enzymatic inactive mutants (H41A and C145A). Cleaved products of Flag-p62-GFP were detected with anti-Flag and anti-GFP antibodies respectively. **h** NSP5 interacted with p62. HEK293T cells were co-transfected with indicated plasmids encoding NSP5 and p62 for 48 h. The cell lysates were prepared for co-immunoprecipitation assays to detect the interaction between NSP5 and p62. **i** NSP5 colocalized with p62. HEK293T cells were transfected with NSP5-GFP and mCherry-p62 and their co-localization was analyzed. Scale bars, 10 μm. **j** Cleavage of recombinant GST-p62 proteins by His-NSP5 in vitro. **k** Schematic picture of the p62 phosphorylation sites and acetylation sites. **l** HEK293T cells were co-transfected with NSP5-Strep and Flag-p62 or its phosphorylation mutants. Cells lysates were evaluated by Western blot. **m** HEK293T cells were transfected with Flag-p62 and its acetylation mutants and NSP5-strep. Cells were then analyzed by Western blot

These results indicated that the reduction in p62 protein levels by NSP5 as independent of the autophagy machinery. Therefore, we hypothesized that the reduction in p62 protein levels indicated the cleavage of p62 by the protease activity of NSP5. We then examined the effect of NSP5 on p62 in HEK293T and HeLa cells. The protein levels of p62 were decreased by NSP5 (Fig. 2a, b). To further investigate whether the decrease in p62 correlated with the protease activity of NSP5, two enzymatically inactive mutants of NSP5 were constructed: NSP5-H41A and NSP5-C145A. The results showed that WT NSP5 decreased p62 protein levels, but NSP5-H41A and NSP5-C145A did not (Fig. 2c, d).

Since the p62 antibody recognizes the full-length p62 protein, it probably cannot detect p62 products after cleavage by NSP5. Therefore, we used exogenous Flag-p62 to examine the cleavage of p62 by NSP5. The results showed that WT NSP5 cleaved p62 in a dose-dependent manner, generating a smaller cleavage product, while the NSP5-H41A and NSP5-C145A mutants could not (Fig. 2e). To further verify the cleaved p62 products, double-tagged p62 (Flag-p62-GFP) was constructed (Fig. 2f). The results showed that anti-Flag and anti-GFP antibodies could detect the cleavage products of p62 (Fig. 2f, g). Co-IP assays demonstrated that NSP5 interacted with p62 (Fig. 2h). P62 and NSP5 colocalized with puncta in the cytoplasm (Fig. 2i). Our results demonstrated that NSP5 cleaved p62 in vivo. We purified His-NSP5 and GST-p62 and performed cleavage assays in vitro*.* The results showed that NSP5 directly cleaved p62 (Fig. 2j). It has been reported that p62 can be modified by phosphorylation and acetylation [35–40]. To explore whether the phosphorylation or acetylation of p62 affects the cleavage of p62 by NSP5, we constructed a series of phosphorylation and acetylation mutants of p62 (Fig. 2k). The results showed that phosphorylation- mimetic mutation on S407 significantly reduced the cleavage of p62 by NSP5, while mutation of other phosphorylation sites (S294E, S349E and S403E) did not affect the cleavage of p62 by NSP5 (Fig. 2l). Acetylation-mimetic mutation of p62 (K295Q, K420Q and K435Q) had no effect on NSP5-mediated cleavage of p62 (Fig. 2m).

It suggests that NSP5 effectively cleaves p62 via the protease activity of NSP5.

### SARS-CoV-2 NSP5 cleaves p62 at residue Q354

**Fig. 3:**
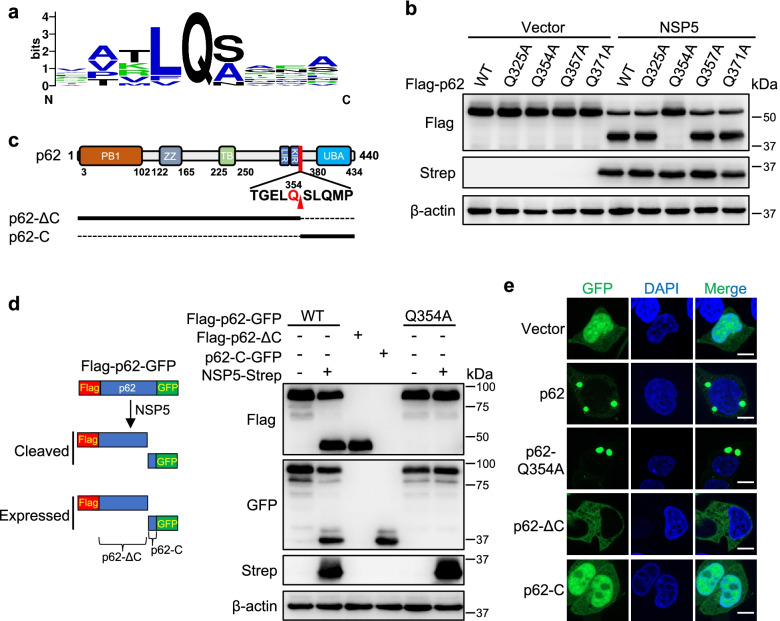
SARS-CoV-2 NSP5 cleaves p62 at residues Q354. **a** Cleavage sites in proteolytic substrates of SARS-CoV-2 NSP5. **b** Potential cleavage sites in p62. HEK293T cells were transfected with NSP5-Strep and Flag-p62 or its mutants. Cells were then analyzed by Western blot. **c** Schematic representation of p62 domain and the cleavage site Q354 by SARS-CoV-2 NSP5. **d** Expected cleavage products of p62 were with similar size as actual cleavage products of p62 by NSP5. Cleavage products of Flag-p62-GFP and Flag-p62-Q354A-GFP were compared with expressed p62 fragments. **e** Cellular localization of GFP-tagged p62 and the indicated mutant or truncates was observed in HEK293T cells. Scale bars, 10 μm

NSP5 cleaves substrate by recognizing the motif X-(L/F/M)-Q↓(G/A/S)-X (where X is any amino acid; ↓ is cleavage site) (Fig. 3a), and the glutamine (Q) residue in the P1 position of the substrate is a crucially conserved cleavage site [41]. Based on the conserved cleavage sites of NSP5, we identified four candidate cleavage sites in p62 (Q325, Q354, Q357 and Q371). To identify the specific cleavage sites in p62, we generated four p62 mutants (p62^Q325A^, p62^Q354A^, p62^Q357A^ and p62^Q371A^). The results showed that the indicated cleavage product of the p62^Q354A^ mutant disappeared in the presence of NSP5, whereas the cleavage products of the other p62 mutants were present (Fig. 3b). The cleavage site Q354 is next to the LIR motif, and cleavage at this site will result in the separation of the PB1, ZZ domains and LIR motif from the UBA domain (Fig. 3c). We constructed two fragments of p62, p62-ΔC and p62-C, to mimic the p62 products produced by cleavage at Q354 (Fig. 3d left). The results showed that the constructed p62 fragments mimicking the p62 cleavage products had the same protein sizes as the actual products of p62 after cleavage by NSP5 (Fig. 3d). Again, the p62^Q354A^ mutant showed resistance to NSP5 cleavage (Fig. 3d). The role of p62 in selective autophagy depends on targeting its cargoes, such as, ubiquitinated protein aggregates, which confers p62 punctate localization (Fig. 3e). The two fragments mimicking the p62 products cleaved at Q354, p62-ΔC and p62-C, showed diffuse localization in the cytoplasm or the nucleus (Fig. 3e).

### Cleavage of p62 by NSP5 disrupts its function in selective autophagy

**Fig. 4:**
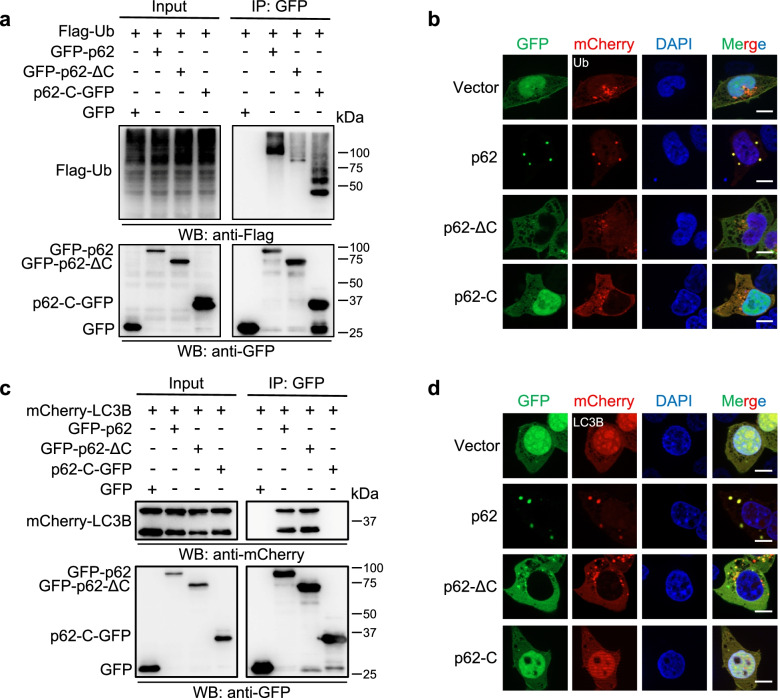
NSP5-mediated p62 cleavage products lost their ability in selective autophagy. **a** HEK293T cells were co-transfected with GFP-p62 or its mutants and Flag-Ub for 48 h. The interaction between p62 or its mutants and Flag-Ub was detected by co-immunoprecipitation assays. **b** Co-localization of GFP-p62 or its truncated mutants and mCherry-Ub was observed in HEK293T cells. Scale bar, 10 μm. **c** HEK293T cells were transfected with GFP-p62 or its truncated mutants and mCherry-LC3 for 48 h. The interaction between p62 or its mutants and mCherry-LC3 was detected by co-immunoprecipitation assays. **d** Co-localization of GFP-p62 or its truncated mutants and mCherry-LC3 was observed in HEK293T cells. Scale bar, 10 μm

p62 functions as a receptor protein to recruit autophagic cargoes into autophagosomes for lysosomal degradation. p62 recognizes ubiquitinated cargoes through its UBA domain and recruits cargoes into autophagosomes by interacting with the autophagosome membrane protein LC3 through the LIR motif [42, 43]. NSP5 cleaves p62 into two parts: the N-terminus (p62-ΔC) contains a LIR motif that binds to LC3, while the C-terminus (p62-C) contains a UBA domain that binds to polyubiquitinated proteins (Fig. 3c). We examined the interaction of p62 cleavage products with ubiquitin and LC3. Co-IP assays showed that intact p62 was capable of binding to both polyubiquitinated proteins and LC3 (Fig. 4a, b), but the binding of p62-ΔC (with LIR and without UBA) to polyubiquitinated proteins was significantly decreased due to the lack of the UBA domain (Fig. 4a), while the binding of p62-C (with UBA and without LIR) to polyubiquitinated proteins was maintained (Fig. 4a). WT and p62-ΔC (with LIR and without UBA) interacted with LC3, while p62-C (with UBA and without LIR) failed to interact with LC3 (Fig. 4c). Consistently, punctate colocalization of WT p62 with ubiquitin or LC3 was not observed in the presence of the p62 cleavage products p62-ΔC and p62-C (Fig. 4b, d).

In brief, NSP5-mediated cleavage of p62 separated the UBA domain and LIR motif, which are important for its role as an autophagy receptor.

### p62 interacts with M protein and mediates its autophagic degradation, which was inhibited by NSP5 cleavage of p62

**Fig. 5:**
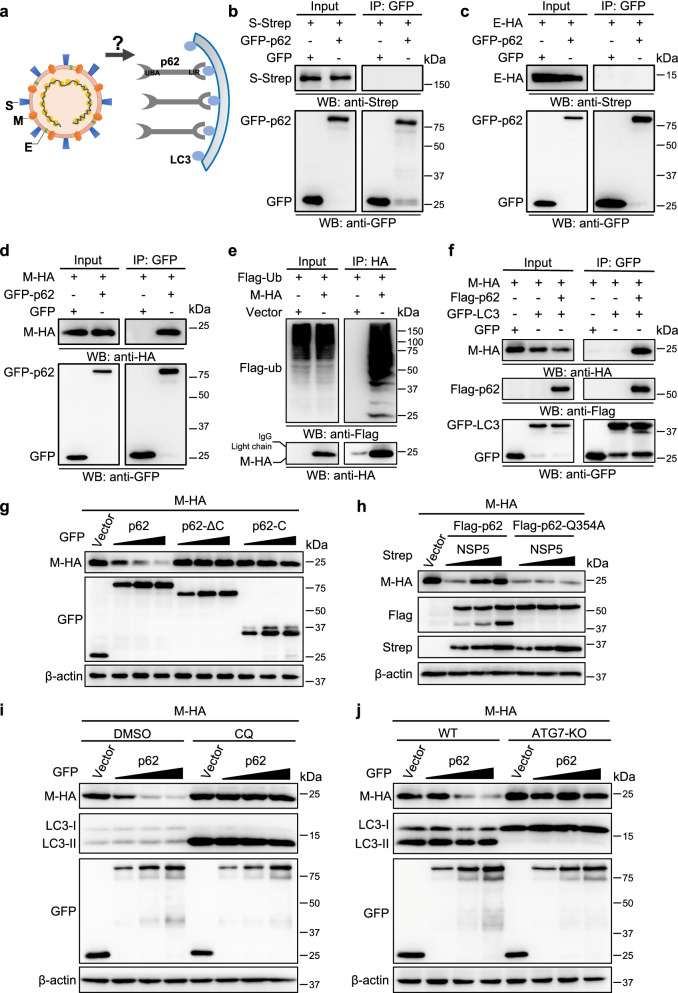
p62 targets SARS-CoV-2 M for autophagic degradation. **a** Schematic diagram of the binding of p62 to SARS-CoV-2 capsid proteins. **b-c** Interaction of p62 with capsid protein S (b) or E (**c**) was analyzed. **d** The SARS-CoV-2 M protein interacted with p62 in HEK293T cells. **e** Ubiquitination of M protein was detected. **f** The interaction of LC3 with M protein was dependent on p62. **g** p62 reduced the protein levels of M protein. **h** NSP5 attenuated the degradation of M protein by p62. **i-j** p62-targeted M degradation was dependent on autophagy. Immunoblotting analysis of HEK293T cells expressed M-HA and GFP-p62 at the presence CQ (**i**) or ATG7 gene knockout (**j**) using the indicated antibodies

As an autophagy receptor, p62 targets intracellular cargoes for autophagosome-lysosome degradation [31, 33, 44]. Therefore, we tried to determine whether this was the case for SARS-CoV-2 and whether p62 bound to SARS-CoV-2 capsid proteins to mediate autophagic degradation. p62 specifically interacted with the M protein, but not the S or E proteins (Fig. 5 a-d). Since p62 binds ubiquitinated cargoes through its C-terminal UBA domain for autophagic degradation, we analyzed whether the M protein was ubiquitinated. M proteins were isolated by immunoprecipitation and then analyzed with ubiquitin antibodies. The results showed heavy ubiquitination of M proteins (Fig. 5e). To evaluate whether p62 could mediate the autophagic degradation of the M protein, we first analyzed the interaction between the M protein and LC3 in the presence of p62. No or little interaction between the M protein and LC3 was observed in the absence of exogenous p62 expression, and the M-LC3 interaction was dramatically increased in cells with exogenous p62 expression (Fig. 5f). This result suggested that the autophagy receptor p62 acts as a bridge to mediate M protein targeting to autophagosomes for autophagic degradation. Indeed, the levels of M protein gradually decreased with increasing expression of p62, and this effect was not observed when the p62 cleavage products p62-ΔC and p62-C were expressed (Fig. 5g). The expression of WT p62 reduced the protein levels of M protein, while further expression of NSP5 blocked this effect (Fig. 5h). The reduction in M proteins induced by the p62-Q354A mutant that resisted NSP5 cleavage could not be blocked by NSP5 (Fig. 5h). These results confirmed that the cleavage of p62 by NSP5 inhibited p62-mediated promotion of the degradation of M protein. To examine whether the degradation of M protein by p62 was dependent on autophagy, we then examined the degradation of M protein under autophagy blockade conditions (autophagy inhibitor CQ or ATG7 gene knockout). The results showed that p62 could not promote the degradation of M protein when autophagy was blocked (Fig. 5i, j), indicating that the degradation of M protein induced by p62 was indeed mediated by the autophagy pathway.

These results revealed that p62 targeted the M protein for autophagic degradation, and this effect was blocked by NSP5 cleavage of p62.

## Discussion

**Fig. 6:**
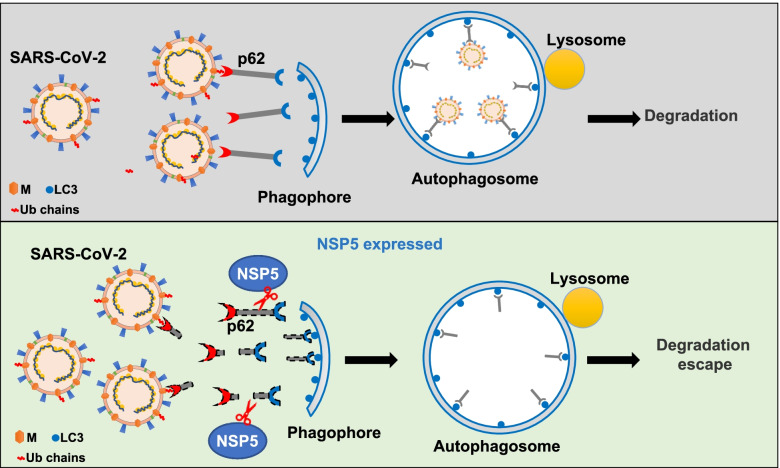
Schematic illustration of SARS-CoV-2 NSP5 cleavage on p62 attenuating p62-mediated autophagic degradation of virus and capsid M protein. Upper, in host cells p62 targets SARS-CoV-2 through recognizing capsid M protein and recruits the virus into double-layered autophagosomes, which eventually leads to lysosomal degradation. Lower, protease NSP5 encoded by SARS-CoV-2 cleaves p62 into two fragments, leading to the separation of functional domains of p62 and loss of ability to mediate selective autophagy of SARS-CoV-2, which causes degradation escape of the virus

Autophagy is the natural immune response of host cells to pathogen infection [45, 46]. Several studies have shown that SARS-CoV-2 infection affects the regulation of autophagy [34, 47–50]. However, there is still a gap between selective autophagy and SARS-CoV-2. We provide evidence that the selective autophagy receptor p62 is cleaved by NSP5, which prevents p62-mediated degradation of the M protein (Fig. 6). Furthermore, p62 cleavage by NSP5 abolishes its ability to mediate selective autophagy. p62 is the key receptor protein that recruits cargos to autophagosomes for lysosomal degradation [51, 52]. p62 can recognize cytosolic cargoes and interacts with LC3 via the LIR motif to target autophagosomes[53]. An interesting finding in this study is that NSP5 cleaves p62 at residue Q354, which separates the UBA domain from the LIR motif (Fig. 3c). Thus, the function of p62 as an autophagy receptor is hampered. Indeed, we found that intact p62 promoted autophagic degradation of the M protein, but the cleaved products of p62 could not (Fig. 5g).

Cleavage of p62 by the encoded proteases of multiple viruses, including seneca valley virus (SVV), coxsackievirus B3 (CVB3), enterovirus D68 (EV-D68) and poliovirus, has been observed [33, 54–56]. The SVV protease 3CL^pro^ targets p62 for cleavage at glutamic acid 355, glutamine 392 and glutamine 395 [33]. The 2A^pro^ protease of CVB3, EV-D68 and poliovirus cleaves p62 at glycine 241, which occurs within its TRAF6-binding domain, impairing the function of p62 in host defense signaling. Although the structures of the proteolytic enzymes encoded by these viruses and the cleavage sites in p62 are different, cleavage of p62 specifically destroys its function and thus helps the virus escape autophagic degradation in the host cell and promotes viral replication. The cleavage of p62 may be a common defense mechanism of these viruses, including SARS-CoV-2 against host cell clearance.

NSP5 also interferes the host cells by cleaving specific proteins. Zhang et al*.* found that NSP5 cleaved RNF20 at Gln521, which prevents degradation of SREBP1 [57]. Fung et al*.* reported that Sendai virus NSP5 inhibits IFN production [58]. Moustaqil et al*.* used an in vitro protease assay to identify target proteins of NSP5 and found that NSP5 cleaved TAB1 and NLRP12 [59]. The researchers also noticed a decrease in TAB1 and NLRP12, suggesting that SARS-CoV-2 infection leads to an imbalance in host innate immunity[59]. These studies showed that NSP5 may help viruses evade the host cell defense mechanisms by cleaving a variety of host cell substrates. NSP5 not only regulates the replication of coronaviruses [60], but also helps viruses escape intracellular degradation by regulating multiple signaling pathways in host cells.

Our study showed that NSP5 cleaves p62 and disrupts the synergistic effect of the functional domains (UBA and LIR) of p62, impairing the receptor function of p62 in the selective autophagic degradation of the SARS-CoV-2 capsid M protein. Our findings uncovered strategies by which SARS-CoV-2 and host cells promote and inhibit clearance of the virus.

## Materials and methods

### Cell culture

HeLa and HEK293T were maintained in DMEM (Invitrogen) supplemented with 10% fetal bovine serum (FBS; Gibco, Life Technologies), 2 mM L-glutamine, and 100U/ml penicillin–streptomycin in a incubator at 37 °C in 5% CO_2_. For treatment of activating autophagy, INK128 (Selleck, S2811) was used at 5 μM for 4 h before harvest. For autophagy blockage, cells were incubated with EBSS supplemented with 1 μg/mL BafA1 for 1 h.

### Plasmids and transfection

The SARS-CoV-2 expression plasmids (all NSP proteins exception of NSP3 and NSP16) were provided by Dr. Nevan J. Krogan (UCSF). The NSP5-GFP were generated by cloning NSP5 into pLVX-EF1alpha-GFP vector. The mutants of NSP5 were generated by cloning NSP5 into pLVX-EF1alpha vector with a 2xStrep tag. cDNAs encoding p62 and its truncated mutants were cloned into pEGFP-C1, pFlag-CMV2 and pGEX-4 T-3 vector, respectively. Site-mutants of p62 were generated by cloning the p62 DNA into pFlag-CMV2 vector. cDNAs encoding M, E and NSP5 were cloned into pLVX-puro vector with a HA tag. The indicated plasmids were transfected using Lipofectamine 2000 (Invitrogen).

### Western blot and antibodies

Cell lysates were harvested and lysed in HU buffer and separated by SDS-PAGE and transferred to PVDF membrane (Bio-Rad). After blocking in TBST containing 5% (w/v) milk for 1 h, PVDF membranes were stained overnight with indicated primary antibodies. Secondary antibodies were incubated and the specific bands were detected. The antibodies were listed as: mouse monoclonal antibody to HA (Santa Cruz Biotechnology, F-7, 1: 5000 for Western blot), mouse Monoclonal antibody to GFP (Santa Cruz Biotechnology, B-2, 1: 5000 for Western blot), mouse monoclonal antibody to FLAG (Sigma, F3165, 1:2000 for Western blot) was from Sigma, mouse monoclonal antibody to Strep (AE066, 1: 5000 for Western blot) and rabbit monoclonal antibody to β-actin (AC026, 1: 10,000 for Western blot) were from ABclonal, rabbit monoclonal antibody to LC3 (Cell Signaling Technology, 4108, 1: 1000 for Western blot), rabbit monoclonal antibody to p62 (Abcam, 109,102, 1: 5000 for Western blot), monoclonal antibody to mCherry (Abbkine, A02080, 1: 5000 for Western blot), monoclonal antibody to GST(Proteintech, 66,001, 1: 5000 for Western blot).

### Purification of His-NSP5 and GST-p62

The His-NSP5 and GST-p62 recombinant proteins were expression in BL21(DE3) *E. coli* cells in LB media at 37 °C. At OD_600_ ~ 1, the cultural temperature was set to 16 °C, and induced with 0.2 mM IPTG. Cells were harvested and then resuspended in lysis buffer. The lysates were centrifuged at 25,000 × *g* for 30 min and purified the soluble protein using gravity flow column packed with Ni–NTA Sefinose or GST-Sefinose. All purified proteins were determined by SDS-PAGE gels.

### Cleavage assays by NSP5 in vitro

For cleavage assays, the recombinant His-NSP5 and GST-p62 proteins were incubated in buffer (20 mM Tris pH 7.8, 100 mM NaCl) at 37 °C for 1.5 h. Cleavage of GST-p62 was analyzed by Western blot, and the His-NSP5 polyacrylamide gels were stained by Coomassie dye.

### Immunefluorescence assays

HEK293T cells were fixed with 4% paraformaldehyde for 10 min at RT and permeabilization in 0.1% Triton X-100 for 20 min. After block with 1% BSA for 30 min, cells were incubated with corresponding primary antibodies followed by incubation with secondary antibodies (Invitrogen, 1:1000 for immunofluorescence). Finally, cells were equilibrated in PBS and 4,6-diamidino-2-phenylindole (DAPI, 0.5 μg/ml) was stained to label the nuclei. Images were obtained on a microscope (Zeiss LSM 880).

### Co-immunoprecipitation assays

For co-immunoprecipitation assays, cells were lysed in lysis buffer (50 mM Tris, 150 mM NaCl, 10% glycerol) supplemented with protease inhibitor cocktail (Roche) for 30 min in ice. Then the supernatants containing rich protein were collected and incubated with GFP-Trap agarose beads, which were followed by washing steps with wash buffer to remove non-specific binding.

### Statistical Analysis

All results were repeated at least three times and representative data or images were shown. Statistical analyses were conducted using GraphPad Prism software (version 6.0). Values in graphs are expressed as means ± SD at least three independent experiments. Differences between different groups were determined for significance two-way ANOVA tests of variance. Significance was set as *p* ≤ 0.05.

## Data Availability

All data needed to evaluate the conclusions in the study are present in this published article.

## Code availability

No applicable.
